# Ex-Vivo Drug-Sensitivity Testing to Predict Clinical Response in Non-Small Cell Lung Cancer and Pleural Mesothelioma: A Systematic Review and Narrative Synthesis

**DOI:** 10.3390/cancers17060986

**Published:** 2025-03-14

**Authors:** Jenny Zipprick, Enes Demir, Hanna Krynska, Sıla Köprülüoğlu, Katharina Strauß, Marcus Skribek, Rita Hutyra-gram Ötvös, Annica K. B. Gad, Katalin Dobra

**Affiliations:** Department of Oncology-Pathology, Karolinska Institutet, 171 77 Solna, Swedenkatalin.dobra@ki.se (K.D.)

**Keywords:** personalized medicine, drug sensitivity testing, 3D-organoid cell culture, targeted treatment, pleural mesothelioma, lung cancer

## Abstract

**Background/objectives**: Non-small cell lung cancer and pleural mesothelioma are among the most lethal and therapy-resistant tumors in humans. These tumors are diagnosed late, frequently present pleural effusion and develop drug resistance, and the treatment is often inefficient and largely alliative. Therefore, there is an urgent need to refine the selection of drugs and patients for personalized treatment. **Methods**: To progress the field, we performed a systematic literature review in line with the PRISMA guidelines followed by a narrative synthesis approach to identify themes. **Results**: The literature to date shows, in general, a positive correlation between the drug-sensitivity of patient-derived cells ex vivo and the clinical outcome. However, only a handful of these studies show a numerical correlation. This, along with the vast diversity of correlated techniques and parameters makes it difficult to directly compare the findings. To build a common knowledge base for future studies, we therefore offer a comprehensive summary of the literature, identify gaps, and suggest future avenues for research. **Conclusions**: We present unified recommendations for the collection, preparation, and ex vivo sensitivity testing of samples for the future development of personalized medicine.

## Simple Summary

Non-small cell lung cancer and pleural mesothelioma are tumors that are diagnosed late, develop drug resistance, and the treatment for them is often inefficient. To contribute to personalized treatments, which offer great potential to improve the outcome of patients with these tumors, we have performed a systematic review of the published literature in the field to assess if drug-sensitivity testing of tumor samples can predict clinical response. We found a positive correlation between the drug-sensitivity of patient-derived cells ex vivo and the clinical outcome. However, our study uncovered methodological inconsistencies, and we identified an urgent need to develop similar protocols and unifying recommendations for drug sensitivity testing. Trends further highlighted that the use of cells derived from pleural liquid present advantages to other approaches.

## 1. Introduction

Lung cancer is a leading cause of cancer-related mortality worldwide, and it is composed of a heterogeneous group of tumors [1,2]. Non-small cell lung cancer (NSCLC) accounts for ~85% of cases, with small-cell lung carcinoma (SCLC) making up the remaining 15% [3,4]. Most lung cancers are diagnosed at an advanced stage [2,5], often with metastases to lymph nodes, pleura, or distant organs, which limits the efficacy of the treatment. The overall 5-year survival rate has long remained around 20% [6]. Nonetheless, recent advances in targeted and personalized therapies have shown promising response rates of up to 80% in selected patients [7], extending survival to approximately 39 months [8]. This highlights that individualized approaches have great potential to improve survival.

Pleural mesothelioma (PM) is another aggressive malignancy, which originates from mesothelial cells lining the pleural cavity [9]. It typically arises in the parietal pleura and spreads to the visceral pleura [10]. Like metastatic NSCLC, PM is often diagnosed late, frequently presenting with pleural effusions, and treatment is largely palliative.

**Figure 1:**
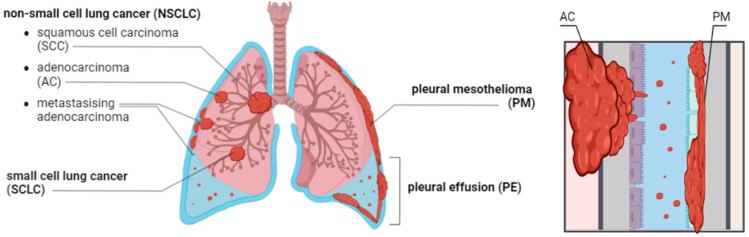
The localization of lung cancer types and their metastatic spreading to the pleura. The pleural cavity with bilateral malignant effusion (blue), bronchial system (light brown), lung (pink), showing the predominant location of tumors (red), specifically various types of NSCLC, i.e., squamous cell carcinoma (SCC), adenocarcinoma (AC), metastasizing adenocarcinoma, along with small cell lung cancer (SCLC), pleural mesothelioma (PM) and pleural effusion (PE), (left), a close up image of AC and PM at the lung periphery (right panel). Lug tissue (pink), interstitial connective tissue (gray), mesothelial lining of the visceral pleura (purple) and parietal pleura (light blue), chest wall (light brown). Created using BioRender.com, Toronto, ON, Canada.

Metastatic NSCLC and PM-related pleural effusions contain shed cancer cells that are morphologically and radiologically indistinguishable (Figure 1). Their diagnosis is further complicated by genetic and morphological heterogeneity, and the diagnosis often requires immunophenotyping and mutation analysis [6]. These challenges underscore the urgent need to improve diagnostic tools and effective strategies for treatments of NSCLC and PM.

Personalized strategies, such as novel targeted therapies, have significantly improved lung cancer treatment over the past decade and hold promise for the future. In a recently developed approach, tumor cells from patient-derived pleural effusions are isolated, followed by ex vivo treatment of the cells with clinically relevant drugs before analysis of cell survival [11]. In this review, the term ex vivo is used to describe the test of drugs on cells in a laboratory environment. Information about the response of the tumor cells to therapy ex vivo allows clinicians to make informed decisions on treatment and to tailor systemic therapy to improve response rates while minimizing toxicity [12]. When combined with DNA sequencing and the characterization of cancer cells, this approach can provide precision medicine with deeper insight into the tumor biology at an individual level. A key advantage of the use of tumor cells from pleural effusions is that their collection via thoracocentesis is a minimally invasive, routine procedure performed to provide diagnosis, alleviate symptoms, and improve quality of life for the patients. Therefore, acquiring cells for ex vivo analysis typically does not add to patient discomfort. Through a systematic review of the literature to date on ex vivo drug responses and how it correlates to clinical outcome, we offer a comprehensive overview of the field. To our knowledge, it is the first systematic review article on this topic. We also explore how ex vivo testing has the potential to transform personalized oncology care and address the practical implications, challenges, and the future steps required to translate this promising technology from bench to bedside.

## 2. Materials and Methods

### 2.1. Search Strategy and Inclusion Criteria

A systematic literature review was carried out in line with Preferred Reporting Items for Systematic reviews and Meta-Analyses (PRISMA) guidelines, followed by a narrative synthesis approach to summarize the results and identify themes. Preregistration-ID: DOI 10.17605/OSF.IO/AB3MF. We used the PubMed (National Center for Biotechnology Information, Rockville Pike, MD, USA) (accession date 31 December 2024), in which we searched the titles and abstracts. Based on the inclusion criteria of lung cancer, in vitro or ex vivo drug testing of patient-derived cells, clinical response of patients to drugs, peer-reviewed original scientific publications in the English language until end of 2024, two reviews, A and B, independently developed the two search queries, as follows.

A. (((((((drug*) OR (chemical compound*)) OR (medicine*)) OR (medication*)) OR (pharma*)) AND ((((((sensitive) OR (sensitivity)) OR (susceptible)) OR (susceptibility)) OR (resistance)) OR (resistant))) AND (“ex vivo” OR “in vitro”)) AND ((cancer [Title/Abstract) OR (adenocarcinom*) OR (carcinom*) OR (tumor*) OR (tumour*)) AND ((lung neoplasms) OR (Pulmonary Neoplasms) OR (Neoplasm*, Lung) OR (Lung Neoplasm) OR (Neoplasm*, Pulmonary) OR (Pulmonary Neoplasm) OR (Lung Cancer*) OR (Cancer*, Lung) OR (Cancer*, Pulmonary) OR (Pulmonary Cancer*) OR (Cancer of the Lung) OR (Cancer of Lung) OR (bronchogenic carcinoma) OR (mesothelioma) OR (pleural mesothelioma) OR (“primary malignant mesothelioma”) OR (adenocarcinom* of lung) OR (lung adenocarcinoma*) OR (lung metast*) OR (pulmonary metast*) OR (pleural effusion*) OR (bronchial metast*) OR ((endobronch*) AND (metast*)) OR ((bronch*) OR (malignant effusion) OR (small cell lung cancer) OR (small cell carcinoma*) OR (non-small cell lung cancer) OR (non-small cell lung carcinoma*) OR (large cell lung cancer) OR (large cell carcinoma*) OR (squamous cell carcinoma*) OR (squamous cell cancer) AND (((patient*) OR (clinic*)) AND ((((survival) OR (outcome)) OR (response)) OR (treatment)))) AND (((((“clinical study”[Publication Type]) OR (“comparative study”[Publication Type])) OR (“evaluation study”[Publication Type])) OR (“journal article”[Publication Type]))) NOT ((head) OR (neck))) NOT (((“meta-analysis”[Publication Type]) OR (“review”[Publication Type])) OR (“systematic review”[Publication Type]))

and

B. (((Drug*) OR (pharmaceutical preparations) OR (Pharmaceutical*) OR (Pharmaceutical Preparation) OR (Preparation*, Pharmaceutical) OR (Preparations, Pharmaceutic) OR (Pharmaceutic Preparations) OR (Pharmaceutical Product*) OR (Product*, Pharmaceutical) OR (Pharma*) OR (medicine) OR (medication)) AND ((((“ex”) AND (“vivo”)) OR (“ex vivo”)) OR ((“in vitro”))) AND ((sensitiv*) OR (drug response) OR (poten*) OR (susceptib*))) AND ((lung neoplasms) OR (Pulmonary Neoplasms) OR (Neoplasm*, Lung) OR (Lung Neoplasm) OR (Neoplasm*, Pulmonary) OR (Pulmonary Neoplasm) OR (Lung Cancer*) OR (Cancer*, Lung) OR (Cancer*, Pulmonary) OR (Pulmonary Cancer*) OR (Cancer of the Lung) OR (Cancer of Lung) OR (bronchogenic carcinoma) OR (mesothelioma) OR (pleural mesothelioma) OR (“primary malignant mesothelioma”) OR (adenocarcinom* of lung) OR (lung adenocarcinom*) OR (lung metast*) OR (pulmonary metast*) OR (bronchial metast*) OR ((endobronch*) AND (metast*)) OR ((bronch*) AND (metast*)) OR (malignant effusions) OR (small cell lung cancer) OR (small cell carcinoma*) OR (non small cell lung cancer) OR (non small cell lung carcinoma*) OR (large cell lung cancer) OR (large cell carcinoma*) OR (squamous cell carcinoma*) OR (squamous cell cancer) NOT ((head) OR (neck))) AND ((patient*) OR (clinic*)) AND ((resistan*) OR (response*) OR (treatment*) OR (therap*) OR (surviv*)) AND ((clinical study[Publication Type]) OR (clinical trial*[Publication Type]) OR (journal article[Publication Type]) OR (comparative study[Publication Type]) NOT (review[Publication Type])) AND (english[Language])

### 2.2. Study Selection

**Figure 2:**
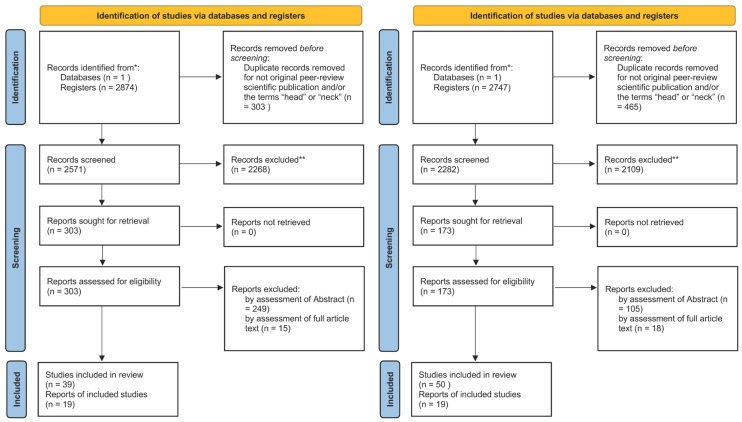
Schematic overview of the literature review process. The two Reviews A (**left**) and B (**right**) were performed independently, based on identical inclusion and exclusion criteria, as described in the Material and Method section, followed by exclusion of a number (n) of publications, as indicated. *: Records identified from PubMed. **: Records excluded after reading the titles.

The two search queries resulted in two lists which thereafter were independently and separately analyzed by the reviewers to identify relevant articles, as shown in Figure 2.

**Figure 3:**
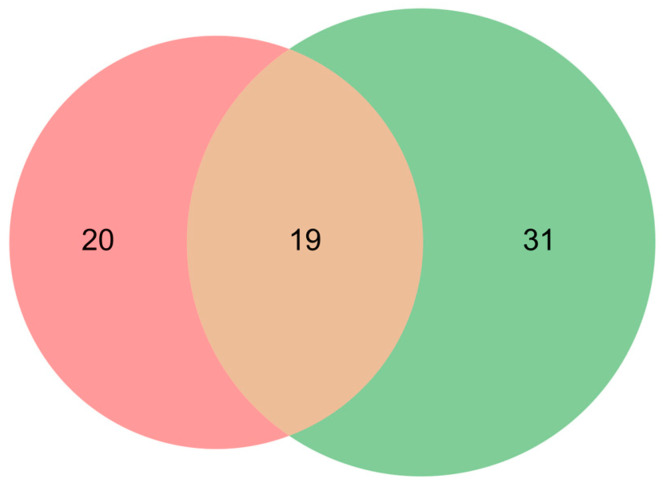
The resulting number of articles identified by two independently developed and performed systematic literature reviews on the same inclusion and exclusion criteria. Articles identified by both review A and B (beige) and only by A (red), or by B (green), as indicated.

The articles independently identified in the review processes A and B (Figure 2) were subsequently reassessed based on inclusion criteria, to ensure a focus on human species and text in the English language. These two independent processes produced two largely overlapping literature lists (Figure 3), which reinforces the validity of the systematic approach. Comparison between the two independent review processes allowed the detection of biases due to missing reports and results. All reports in the lists were then analyzed again, regarding if they showed the data of interest, i.e., ex vivo and clinical data from same patients. We found that only the 19 overlapping reports presented this data, which shows the robustness of the approach, and provides confidence in the body of evidence.

### 2.3. Data Extraction

The following data were thereafter extracted from the 19 publications: Article title, name of first author, origin of cells used, number of patients, ex vivo cell culture model, method of analysis of ex vivo outcome, the method used to correlate ex vivo data to the response of patients in the clinic, and the statistical significance of this correlation, and the drugs used for the comparison between ex vivo and clinical data.

### 2.4. Thematic Analysis

The extracted data from the results sections of the various studies were aggregated, and the findings were summarized, critically analyzed, compared, and organized so that common denominators and themes could be delineated. We focused on the origin of the patient-derived cells, number of patients, cell culture model used, the ex vivo and clinical parameters correlated, if a correlation was found, the correlation value, and statistical method used for the correlation. Due to lack of standardized and comparable numerical outcomes, the thematic analysis was not followed by a numerical meta-analysis, such as meta-regression. Thereafter, the most-used concepts, terminology, and definitions, gaps in the literature, challenges, and future avenues for science were identified.

## 3. Results

For several decades, patient-derived cancer cells have been tested ex vivo to predict the response of patients to the drugs in the clinic. The earliest investigations focused on NSCL and took place more than 30 years ago. Despite the promising potential of ex vivo drug-testing, the field has been challenged by difficult techniques which require development and refinement, non-standardized methods and validation procedures, and a lack of common conceptual framework and definitions. To date, none of the established methodologies have been successfully incorporated in the clinical workflow. We have here performed a systematic literature review, in which we analyzed almost three thousand publications, and selected and extracted information from 19 articles, as described in the Methods section, and presented in Table 1. We could thereby identify common themes in the research literature to date on the ex vivo drug-sensitivity testing of patient-derived cells from lung cancer and mesothelioma patients, as follows.

### 3.1. Historical Progress Towards the Use of 3D Multicellular Model Systems with the Aim to Mimic the Papillary Growth of Cancer Cells In Vivo

Our systematic literature review shows how the research field has evolved over the past three decades. While early studies investigated both SCLC and NSCLC, recent research has predominantly focused on NSCLC. The early studies aim to establish and use cell lines from biopsy tissues, whereas more recent studies culture patient-derived cells (PDC) in adherent 2D cultures. These 3D cell aggregates are often called spheroids due to their circular shape or tumoroids. Even though a tumor is not an organ, they are even sometimes referred to as organoids. With time, we see a growing emphasis on the culture of the patient cells in suspended 3D cultures. The culture of patient cells in 3D, which allow cell–cell contacts and signaling in suspension, better mimic the in vivo situation than the earlier models. Recent findings that 2D cultures show greater sensitivity to drugs, compared to 3D organoids [13], highlight the importance of using cell culture conditions that mimic the in vivo situation as closely as possible. This discrepancy observed between the 2D, and 3D culture has been suggested to be due to the fact that 3D models more effectively mimic the environment of the tumor cells in the patient [14,15,16,17]. In this context, it is of interest to note that cells in the PE grow in 3D aggregates in which the cells are loosely connected in a non-spherical structure, and when in solution ex vivo, the PE-derived cells form similar aggregates. Taken together, this suggests that PE-based cell sensitivity testing can allow better prediction than 2D surfaces and 3D spheroids.

### 3.2. Ex Vivo Drug-Sensitivity Identifies Optimal Therapy, Suggests More Effective Treatments, and Prevents the Use of Drugs to Which the Tumor Is Resistent

Drug resistance is a major obstacle to effective therapy of NSCLC, and in most patients, it is the main reason for treatment failure. The underlying cause of drug resistance is that in a large population of genetically unstable cancer cells, the selective pressure favors the survival of cells with mutations that provides drug-resistance. Drug resistance hampers the efficacy of successful therapy and leads to the progression of the disease. Because the consequences of inappropriate medications are severe, both regarding high cost and toxicity, it is crucial to find the optimal therapy for everyone. Our literature review shows that clinically relevant, effective assay systems can shed light on drug-resistance mechanisms and allow the identification of options for optimal drug combination before treatment. The study by Wu et al. [18] highlights the potential of using conditional reprogramming (CR) technology to culture cancer cells from pleural effusions in advanced NSCLC patients to overcome the challenges of primary resistance in the treatment. This study focuses on cases of NSCLC with primary resistance to epidermal growth factor receptor tyrosine kinase inhibitors (EGFR-TKI) therapy and highlights the failure of TKIs as effective treatments. They found a strong correlation between the sensitivity results obtained in vitro and clinical response for drug combinations, specifically for cisplatin and pemetrexed. Hence, they provide validation for the predictive use of CR-derived drug sensitivity tests. The study also emphasizes that CR can guide tailored therapies for patients with TKI-resistant lung cancer and significantly improves patient outcome. By use of an ATP-based tumor chemosensitivity assay (ATP-TCA), Chen et al. [19] evaluated the in vitro chemosensitivity to eight single-drug chemotherapies of 120 lung cancer patients and the multi-drug resistance (MDR) to seven chemotherapy regimens of 291 lung cancer patients. They provided evidence that ATP-TCA is a robust tool to predict the clinical response of lung cancer. They found that the level of chemosensitivity varies greatly across different drugs and combinations and that paclitaxel and cisplatin demonstrate the highest efficacy. In addition, they observed a high prevalence of multi-drug resistance in lung cancer. This study demonstrates the importance of pre-treatment sensitivity testing in lung cancer to provide the selection of personalized regimens and to minimize the risk of ineffective treatments. The study by Karekla et al. [20] further shows that resistant tumor cells accumulate less drug, and that tumors with TP53 mutations are more sensitive to cisplatin. They further show how explant cultures can preserve the tumor microenvironment and present a correlation between ex vivo cisplatin sensitivity and patient survival.

While cost-effectiveness is an important consideration in the clinical implementation of ex vivo drug sensitivity testing, formal economic evaluations require dedicated health economic modeling, which varies by healthcare system and reimbursement policies. Future studies assessing the financial impact and comparative cost-effectiveness of ex vivo testing versus molecular diagnostics, such as NGS and ctDNA analysis, are warranted to inform policy decisions.

### 3.3. Ex Vivo Drug Sensitivity Results Can Predict Patient Response and Survival

All publications that we identified in our literature review presented details on the drug concentrations used in the ex vivo drug-sensitivity testing (DST) as well as in the clinic. These were in the same range and based on standard therapeutic concentrations. The majority (16) of the 19 articles identified suggest a general positive correlation between the outcome of ex vivo DST of patient-derived cells and the outcome of the patients in the clinic. Four articles observed no correlation. Although the major part (13) of these 16 studies presented numerical values of the correlation, 6 instead presented non-numerical, descriptive qualitative analyses.

While various parameters were analyzed for patient outcome in the clinic, the main parameters were overall survival, which is the time that the patient is alive from the date of diagnosis or start of treatment, and progression-free survival, the time from random assignment in clinical trials to disease progression or death.

Moon et al. [21] evaluated 34 patients with unresectable NSCLC via ATP-based chemotherapy response assay to assess 34 patients with unresectable NSCLC and classified 20 patients as platinum-sensitive. This platinum-sensitive group showed a higher clinical response rate of 61.1% as compared to 21.4% in the platinum-resistant group (*p* = 0.036). Furthermore, patients in the sensitive group experienced prolonged median progression-free survival (3.9 months vs. 3.3 months) and overall survival (13.2 months vs. 8.5 months), though only overall survival was statistically significant (*p* = 0.025). Although these results highlight the clinical utility of the assay, the relatively short median progression-free survival underscores the limitations of platinum-based regimens.

The study conducted by Szulkin et al. [22] confirmed that patients with higher overall chemosensitivity experienced longer progression-free and overall survival, emphasizing the assay’s predictive value. The results of Vinayanuwattikun et al.’s [23] study showed that treatment plans guided by in vitro data, such as the use of gemcitabine or vinorelbine, improved progression-free survival in some cases.

Inoue et al. [24] evaluated adjuvant chemotherapy options in stage IB-IIIA NSCLC patients after complete surgical resection with the collagen gel droplet-embedded culture drug sensitivity test (CD-DST). Among 75 patients successfully tested, 76% demonstrated sensitivity to carboplatin and/or paclitaxel. Of these, 30 patients received adjuvant therapy based on CD-DST results, achieving a 5-year OS rate of 82% and a DFS rate of 68%. In contrast, chemotherapy resistant patients had lower survival outcomes, although differences did not reach statistical significance due to the small sample size. While the CD-DST successfully identified patients likely to benefit from chemotherapy, the study noted no significant survival difference between sensitive and non-sensitive groups when analyzed in broader cohorts.

Taken together, our literature review reveals a growing trend over time to explore the correlation between ex vivo drug sensitivity and patient response. While early studies by Shaw et al. in 1993 [25] and 1996 [26] did not find a significant correlation, more recent publications show a clear correlation. For instance, Shie et al. in 2023 reported an accuracy of 85%, a sensitivity of 86.7%, and a specificity of 80% when culturing PDC in spheroid-like 3D cultures [27]. However, suitable assay systems and an adequate sample size are required, and the clinical use remains unclear.

### 3.4. Development of 3D Ex Vivo Cell Culture Models with Extracellular Matrix Components to Better Mimic the Tumor Microenvironment

The extracellular environment around cancer cells often suppresses cancer; however, it can switch into a tumor-promoting tumor microenvironment. This microenvironment mainly consists of the proteins of the extracellular matrix (ECM), water and non-cancer cells, such as cancer-associated fibroblasts, endothelial, and immune cells. To mimic this microenvironment, many 3D cell culture models identified in our literature review added collagen or lung extracellular matrix to the spheroids. However, we could not detect a clear improvement in the predictive ability of the models, as compared to 3D models with no matrix added (Table 1). Nonetheless, model systems that mimic the complex microenvironment of the tumor stroma remain of key interest to the research field.

Shie et al. [27] designed an ex-vivo lung cancer spheroid model that integrates patient-derived tumor cells with a decellularized extracellular matrix from the lung to replicate native tumor microenvironments. The spheroid model was engineered to recapitulate features of the tumor microenvironment such as hypoxia and vasculature. In a co-clinical trial involving 20 patients, the model achieved high predictive accuracy (85%) for clinical responses to chemotherapeutics and targeted therapies. The model revealed consistent sensitivity patterns, with high specificity for resistant and non-resistant cell lines, including EGFR-mutant cases. For example, gefitinib-resistant spheroids demonstrated drug responses that reflected patient outcomes.

Karekla et al. studied whether explant cultures provide evidence of their utility in predicting patient responses to chemotherapeutic agents [20]. By maintaining the tumor microenvironment, the sensitivity to cisplatin, which was observed in 57% of cases, correlated to patient survival, independent of tumor stage. Explant cultures preserve the native tumor microenvironment, which offers potential for model systems for drug testing that better mimics the tumor microenvironment in tissue.

### 3.5. Cancer Cells from Pleural Effusions Show Growth Advantages Ex Vivo, as Compared to Cells from Biopsies

**Figure 4:**
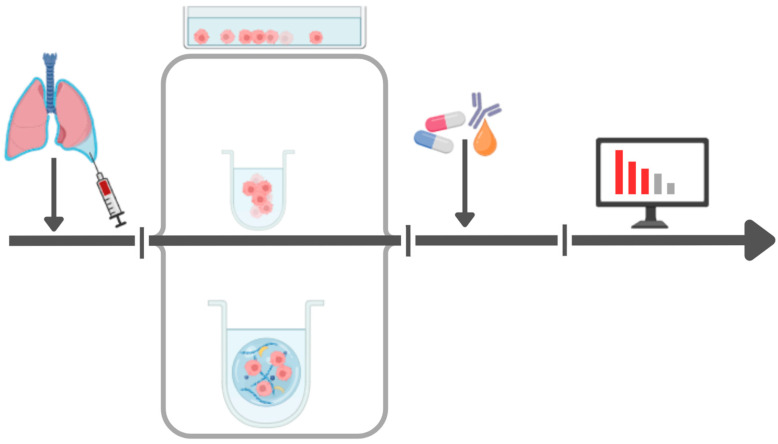
The stages and cell model systems of the ex vivo sensitivity testing process: from left to right, sample acquisition from patient by thoracocentesis, cell culture ex vivo; (from top) in a 2D monolayer; (middle) in cell aggregates in a suspension that mimic cell aggregates in PE; (bottom) cell spheroid, organoid or tumoroid, showing cancer cells (pink), immune cells (small dark blue), fibroblasts (yellow), and extracellular matrix components (blue and light blue lines), followed by drug treatment, immunostaining data acquisition and analysis, as indicated. The literature review highlights the many advantages of using PE-derived cells and –cell culture model systems.

Today, studies use tissue samples from biopsies as well as cells derived from pleural effusions. Additionally, some research has involved xenografts, where fresh tumor pieces are implanted into immunodeficient mice for in vivo testing. While xenograft models are valuable for the preclinical evaluation of new cancer therapies, they have a low success rate for establishing viable cultures, requiring 2 to 12 months, and incurring significant costs [28,29,30]. A major challenge in these studies has been the establishment of viable cell cultures, as sensitivity testing cannot be performed without them. However, the success rate for creating these cultures has improved over the last 30 years. Some studies suggest that the success rate for establishing primary cultures from pleural effusion samples is significantly higher than for solid tumor samples, with success rates exceeding 90% [29]. This higher success rate is attributed to the low number of cancer cells found in solid tumor samples, which are often outgrown by stromal cells, particularly stromal fibroblasts [31]. In summary, this highlights the possibility that the use of pleural effusion-derived patient cancer cells and cell culture models can have a higher predictive strength than cancer cells from other tissue and analyses based on other types of model systems (Figure 4).

**Table 1:** Original scientific publications describing the use of ex vivo drug-sensitivity tests of patient-derived cells to predict the effect of treatment on non-small cell lung cancer and mesothelioma patients. Publications are shown in an inverse chronological order, with information on the number of patients, origin of patient-derived cells (PDC), cell culture model with added ECM/coating if indicated, when applicable, ex vivo and clinical parameters correlated, correlation observed, and statistical method used. For each study, we also show the treatment used in the clinic. Acronyms are as follows, pleural effusion (PE), two dimensional (2D) and three dimensional (3D) cell culture, Poly-L-ornithine (PO), Poly-L-lysine (PLL), lung decellularized extra cellular matrix (LdECM), cell adhesive matrix (CAM), drug sensitivity testing (DST), patient-derived xenograft (PDX), progression-free survival (PFS), overall survival (OS), disease-free survival (DFS), median survival time (MST), clinical response rate (CRR), partial response (PR), stable disease (SD), progressive disease (PD), Therapy response (TR), Docetaxel (TXT), versus (vs), Erlotinib (ERL), Gefitinib (GEF), Afatinib (AFA), Pemetrexed PEM), Gemcitabine (GEM), Icotinib (ICO), Cisplatin (Cis), Docetaxel (DTX), Carboplatin (CAR), Vinorelbine (VNR), Paclitaxel (PTX), Osimertinib (OS), Entrectinib (ENT), Crizotinib (CRZ), Atezolizumab (ATEX), Doxorubicin (DOX), Oxaliplatin (OXA), Etoposide (ETO), Vincristine (VCR), Vindesine (VDS). Cyclophosphamide (CTX), Vinblastine (VBL), Mitomycin C (MMC), Cyclophosphamide (CPP), 5-fluorouracil (5-FU), Lomustine (CCNU), 4-hydroperoxycyclophosphamide (4-HC), Carmustine (BCNU), Bleomycin (BLM).

Reference	Number of Patients	Cell Origin	Cell Culture Model	Ex Vivo and Clinical Parameters Correlated	Observed Correlation Between Ex Vivo and Patient Response (Correlation Value)	Statistical Method	Patient Treatment in Clinic
Shie et al., 2023 [27]	20	AC, SCLC (biopsy)	2D, 3DLdECM	DST vs. TR	Yes (85% accuracy ^1^)	N/A	ERL, GEF, AFA, PEM, GEM
Wu et al., 2020 [18]	1	NSCLC (TKI-resistant) (PE)	2D PO	DST vs. TR	Yes, qualitative (N/A)	N/A	ICO, Cis, GEM, PEM, DTX
Papp et al., 2020 [32]	14	AC (biopsy, PE)	3D	DST vs. TR	Yes (93% accuracy ^1^)	N/A	Cis, CAR, VNR, GEM, PTX, PEM, ERL, GEF
Kim et al., 2019 [33]	10	AC (biopsy, PE)	2D collagen IV	DST vs. TR, PFS	Yes (100% accuracy ^1^)	N/A	OS, GEF, ENT, CRZ
Vinayanuwattikun et al., 2019 [23]	11	NSCLC (PE)	2D	DST vs. TR	Yes, qualitative (N/A)	N/A	TXT, GEM, ERL, PEM, VNR, CAR, PTX
Hillerdal et al., 2017 [11]	8	AC, PM (PE)	3D	DST vs. TR	Yes (78% accuracy ^1^)	N/A	CAR, Cis, GEM, DOX, PEM, VNR
Chen et al., 2018 [19]	24	Lung cancer tissue	2D	PFS and OS of DST sensitive vs. resistant	Yes (overall), *p* = 0.046 (PFS), *p* = 0.036 (OS) (TXT only), *p* = 0.041 (PFS), *p* = 0.040 (OS)	Wilcoxon	Cis, PEM, OP, EP, CAR, VNR, VNC, TXT, GEM, PTX
Karekla et al., 2017 [20]	25	NSCLC (biopsy)	2D	DST vs. MST	Yes, (*p* = 0.019)	Kaplan–Meier, Cox regression	Cis
Inoue et al., 2018 [24]	75	NSCLC (biopsy)	3D collagen I	DST sensitive vs. 5-year OS, DFS	Yes, 5-year OS 82% (*p* = 0.039); DFS 68% (*p* = 0.089)	Kaplan–Meier, Wilcoxon	CAR, PTX
Roscilli et al., 2016 [34]	6	AC (PE)	2D	DST vs. TR	Yes (100% accuracy ^1^)	N/A	Cis, CAR, TXT, VNR, GEM, GEF, ERL
Szulkin et al., 2014 [22]	16	PM, healthy patient (PE)	2D	DST sensitive vs. OS	Yes (*p* = 0.005)	Unpaired *t*-test	31 different, e.g., Cis, CAR, GEM, PEM, PTX, DOC, VNC, VNR, EP
Higashiyama et al., 2010 [35]	81	NSCLC (biopsy)	3D collagen I	DST vs. TR	Yes (70% accuracy ^1^)	N/A	Cis, CAR, PTX, DOC, VNR, GEM
Kawamura et al., 2007 [36]	49	Metastatic biopsies, PE	3D collagen I	DST sensitive vs. MST	Yes (*p* = 0.027)	Kaplan–Meier, Cox regression	DOC, PTX, CPT-11, VNR, GEM, Cis, CAR, VDS, EP
Moon et al., 2007 [21]	34	NSCLC (biopsy)	2D	CRR, PFS ^2^, OS of DFS sensitive vs. resistant	CRR *p* = 0.036 PFS *p* = 0.060 OS *p* = 0.025	Kaplan–Meier, Cox regression	Cis, CAR, PTX, DOC, GEM, VNR
Higashiyama et al., 2001 [37]	25	NSCLC	3D collagen I	DST vs. TR	*p* = 0.001	Chi-squared test	Cis, CAR, ETO, 5-FU, MMC, VDS
Shaw et al., 1996 [26]	21	NSCLC (lung, metastatic biopsy, PE)	2D	DST sensitive vs. CRR	CRR *p* = 0.86 OS *p* = 0.34	Fisher’s exact	12 different drugs, e.g., Cis, EP, CTX, VNC, …
Shaw et al., 1993 [25]	90 ^3^	NSCLC (lung and metastatic biopsy)	2D	DST sensitive vs. CRR, OS of DST sensitive vs. resistant	CRR *p* = 0.076 OS *p* = 0.34	Fisher’s exact test, Kaplan–Meier, Mantel-Haenszel	12 different, e.g., Cis, EP, VNC, DOX, VBL, MM-C, …
Wilbur et al., 1992 [38]	25	NSCLC (tissue, PE)	3D	DST sensitive vs. CRR	Yes (*p* = 0.04)	Wilcoxon	Cis, CPP, DOX, EP, 5-FL, IM, MM-C, VBL, VNC
Ajani et al., 1987 [39]	14	Lung (tissue, PE)	2D CAM	DST vs. CRR	Yes (93% accuracy ^1^)	N/A	DOX, 4-HC, 5-FU Cis, EP, MM-C, VNC, BCNU, BLM

^1^ Accuracy, as the fraction matching ex vivo data versus clinical results. ^2^ Only 30 patients have been included in this analysis. ^3^ Only 21 patients were used for the DST vs. CRR study.

## 4. Discussion

Personalized medicine bridges the gap between in vitro and ex vivo experimental models and real-world clinical outcomes by the direct correlation of the ex vivo responses of patient-derived cells with the response to the same treatments by the patient. Our literature review that specifically focusses on lung cancer and mesothelioma demonstrates several limitations of the approach, such as small sample sizes and the lack of consistency in the parameters evaluated to establish correlations, which underscore the difficulty of translating ex vivo findings into clinical benefit. The diverse model systems used for ex vivo cell culture and growth of patient-derived cancer cells also present challenges. For all studies, it is instrumental that cancer cells respond to drugs ex vivo in a similar way as in the patient. Our review indicates that similar results are obtained in 2D and 3D models of cancer. Nonetheless, 3D models have been shown to better reflect cancer characteristics such as oxygen gradient and necrosis in the core of compact tumor cell clusters, which highlights that 3D models can be more physiologically relevant models [40]. Cells that grow in 2D appear to be more sensitive to drugs, because the drugs do not need to penetrate into a structure to reach cancer cells.

Our systematic literature review showed that the studies in which the ex vivo data did not show a positive correlation to patient outcome were based on tissue biopsies of NSCLC. This could be related to the sample preparation and the number of passages of the cells prior to the DST, as we could observe it in their protocol section [25]. This may also suggest that it is more difficult to mimic the environment of a solid tissue, from which biopsies are taken, than the growth conditions in PE, in which the cells grow as aggregates in suspension. Although research in ex vivo testing of lung cancer and mesothelioma cells switched from 2D to 3D cell culture systems during the last decades, it is not clear to which extent this has increased the correlation between the ex vivo response and the outcome of patients in the clinic. This highlights that much remains unknown about how the microenvironment in which normal and cancer cells grow influences the behavior of cells. For example, in the body, cancer cells in solid tissue can bind to remaining basal lamina or to collagen fibers of a similar width as a cell, which both would present to the cell as a stiff 2D surface and not a 3D environment. In addition to spatial cues, many more parameters in the cell environment regulate cell behavior, including the stiffness, degradation, and relaxation of, and fiber width within the ECM, as well as the chemical composition and crosslinking of this matrix [41,42,43,44]. Also, non-cancer cells in the tumor microenvironment, such as immune cells and fibroblasts exert an instrumental control on the function of cancer cells [45,46]. We speculate that the lack of correlation in the study of Shaw’s lab [25,26] where cells from tissue were used is because it is more difficult to mimic the complex tumor environment in the tissue, with active immune and stromal response than the cell aggregates in the PE. The observation that the addition of the ECM component collagen to 3D cell culture models did not provide a predictive advantage of the models is in line with the concept that it is difficult to mimic the tumor microenvironment. Very recently, techniques that allow analysis of both cancer- and immune cells in 3D have been developed [47,48]. In the future, these and similar techniques that mimic the immune system in the microenvironment can provide an opportunity to predict patient response to immune checkpoint inhibitors in the clinic. Given the status of immunotherapy as the standard of care, this is a promising area for future research. However, there is currently no evidence that increasing the complexity of the model, such as organoid, may provide a more accurate predictive strength or would present cost- and time-advantages easily includable in clinics. A compromise should be found between the accuracy of the approach, time, and cost and the qualitative response prediction of the patient to the therapy. Nonetheless, as cancer cells in the PE of the patient grow in multi-cellular 3D aggregates in a liquid and are therefore less exposed to the complex signals by extracellular matrix proteins, fibroblasts, and immune cells than in tissue, we expect the PE environment to be simpler to mimic ex vivo than tumor microenvironments of solid tissues. This highlights that a focus on PE-derived cells can provide an important avenue for future research into the ex vivo DST research field.

We show that a large variety of different approaches has been used to analyze patient response in the clinic, including overall survival, clinical response rate, progression-free survival, median survival time, and clinical response, which all appeared to provide predictive quality data. A total of 8 of 19 articles identified in our systematic literature search showed numerical values of the correlation between the ex vivo data to the clinical outcome of patients, 6 instead presented qualitative comparisons. For the articles that showed numerical correlations, it is difficult to directly compare these to each other, as relevant data were obtained by different experimental approaches and different statistical methods were used to quantify the correlations. Taken together, this indicates that there is an urgent need for the field to decide on the same clinical outcome and the statistical approaches to use for numerical correlations.

Together with the recent development of high-throughput screening technologies such as automated microfluidic systems and single-cell analysis, ex vivo sensitivity testing has great potential to improve treatment efficiency and to allow integration of genomic data for personalized treatment. In the future, a detailed characterization of the tumor on DNA and proteome level should be an integral part of functional ex vivo sensitivity testing, as it can provide important insights into targetable mutations and drug-resistant phenotypes. Ex vivo DST helps to select the most effective drug for each patient, thereby improving treatment efficiency. Ideally, the ex vivo testing should be combined with a detailed molecular analysis to optimize the conditions for personalized medicine. This approche would reduce the risk of unnecessary delay in patient management by identifying the most effective drug combinations on an individual basis. Ex vivo DST has potential economic implications. Its ability to provide real-time, functional drug response data could reduce reliance on broad-panel sequencing, decrease the number of ineffective treatments, and ultimately optimize resource allocation. However, these hypotheses require dedicated cost-effectiveness studies incorporating real-world clinical and economic data, which are beyond the scope of our current review. Further studies assessing the financial impact of ex vivo DST within different healthcare settings are warranted.

Our review highlights the urgent need for standardized procedures and unifying principles for lung cancer and mesothelioma-derived DST. These are principles that would allow direct comparisons between reports and conclusion, allow synthesis of a knowledgebase, and that could promote the field towards clinical use. For this, we suggest that future studies on ex vivo DST of lung cancer and mesothelioma include information about key clinical, stage, grade, prior treatment regimen, and driver mutations of the tumors. The literature calls for studies that use ex vivo cell culture systems that more closely mimic the in vivo tumor microenvironment in terms of chemical, physical, spatial, and cellular parameters. In addition, we identified a need to present numerical correlation values between ex vivo data to the outcome of patients in the clinic, and to use the same patients or patient cohort for the correlation between ex vivo drug-response data and the patient outcome.

## 5. Conclusions

Taken together, this literature review calls for unified principles and procedures in the field of ex vivo drug-testing of patient-derived cells from lung cancer and mesothelioma. We identify gaps in the knowledge, present unifying suggestions, and recommendations for future studies, and suggest novel avenues for research, e.g., we highlight the advantages of high-throughput analysis of PE-derived cells, which holds great promise for the future. It is our hope that this study will promote the urgently needed new personalized treatments for a radically improved clinical outcome for patients with lung cancer or mesothelioma.

## References

[1] Travis W.D., Brambilla E., Burke A.P., Marx A., Nicholson A.G. (2015). Introduction to The 2015 World Health Organization Classification of Tumors of the Lung, Pleura, Thymus, and Heart. J. Thorac. Oncol..

[2] Bray F., Laversanne M., Sung H., Ferlay J., Siegel R.L., Soerjomataram I., Jemal A. (2024). Global cancer statistics 2022: GLOBOCAN estimates of incidence and mortality worldwide for 36 cancers in 185 countries. CA Cancer J. Clin..

[3] Howlader N., Forjaz G., Mooradian M.J., Meza R., Kong C.Y., Cronin K.A., Mariotto A.B., Lowy D.R., Feuer E.J. (2020). The Effect of Advances in Lung-Cancer Treatment on Population Mortality. N. Engl. J. Med..

[4] Zappa C., Mousa S.A. (2016). Non-small cell lung cancer: Current treatment and future advances. Transl. Lung Cancer Res..

[5] Ferlay J., Colombet M., Soerjomataram I., Parkin D.M., Pineros M., Znaor A., Bray F. (2021). Cancer statistics for the year 2020: An overview. Int. J. Cancer.

[6] Jeon D.S., Kim H.C., Kim S.H., Kim T.J., Kim H.K., Moon M.H., Beck K.S., Suh Y.G., Song C., Ahn J.S. (2023). Five-Year Overall Survival and Prognostic Factors in Patients with Lung Cancer: Results from the Korean Association of Lung Cancer Registry (KALC-R) 2015. Cancer Res. Treat..

[7] Soria J.C., Ohe Y., Vansteenkiste J., Reungwetwattana T., Chewaskulyong B., Lee K.H., Dechaphunkul A., Imamura F., Nogami N., Kurata T. (2018). Osimertinib in Untreated EGFR-Mutated Advanced Non-Small-Cell Lung Cancer. N. Engl. J. Med..

[8] Ramalingam S.S., Vansteenkiste J., Planchard D., Cho B.C., Gray J.E., Ohe Y., Zhou C., Reungwetwattana T., Cheng Y., Chewaskulyong B. (2020). Overall Survival with Osimertinib in Untreated, EGFR-Mutated Advanced NSCLC. N. Engl. J. Med..

[9] Yang H., Testa J.R., Carbone M. (2008). Mesothelioma epidemiology, carcinogenesis, and pathogenesis. Curr. Treat. Options Oncol..

[10] Yap T.A., Aerts J.G., Popat S., Fennell D.A. (2017). Novel insights into mesothelioma biology and implications for therapy. Nat. Rev. Cancer.

[11] Hillerdal C.-O., Ötvös R., Szatmári T., Own S.A., Hillerdal G., Dackland Å.-L., Dobra K., Hjerpe A. (2017). Ex Vivo Evaluation of Tumor Cell Specific Drug Responses in Malignant Pleural Effusions. Oncotarget.

[12] Ötvös R., Szulkin A., Hiilerdal C.-O., Celep A., Yousef-Fadhel E., Skribek H., Hjerpe A., Szekely L., Dobra K. (2015). Drug Sensitivity Profiling and Molecular Characteristics of Cells from Pleural Effusions of Patients with Lung Adenocarcinoma. Genes Cancer.

[13] Mazzocchi A., Devarasetty M., Herberg S., Petty W.J., Marini F., Miller L., Kucera G., Dukes D.K., Ruiz J., Skardal A. (2019). Pleural Effusion Aspirate for use in 3D Lung Cancer Modeling and Chemotherapy Screening. ACS Biomater. Sci. Eng..

[14] Akerlund E., Gudoityte G., Moussaud-Lamodiere E., Lind O., Bwanika H.C., Lehti K., Salehi S., Carlson J., Wallin E., Fernebro J. (2023). The drug efficacy testing in 3D cultures platform identifies effective drugs for ovarian cancer patients. NPJ Precis. Oncol..

[15] Kaur G., Doroshow J.H., Teicher B.A. (2021). Format (2D vs 3D) and media effect target expression and response of patient-derived and standard NSCLC lines to EGFR inhibitors. Cancer Treat. Res. Commun..

[16] Murakami S., Tanaka H., Nakayama T., Taniura N., Miyake T., Tani M., Kushima R., Yamamoto G., Sugihara H., Mukaisho K.I. (2021). Similarities and differences in metabolites of tongue cancer cells among two- and three-dimensional cultures and xenografts. Cancer Sci..

[17] Tidwell T.R., Rosland G.V., Tronstad K.J., Soreide K., Hagland H.R. (2022). Metabolic flux analysis of 3D spheroids reveals significant differences in glucose metabolism from matched 2D cultures of colorectal cancer and pancreatic ductal adenocarcinoma cell lines. Cancer Metab..

[18] Wu M., Hong G., Chen Y., Ye L., Zhang K., Cai K., Yang H., Long X., Gao W., Li H. (2020). Personalized drug testing in a patient with non-small-cell lung cancer using cultured cancer cells from pleural effusion. J. Int. Med. Res..

[19] Chen Z., Zhang S., Ma S., Li C., Xu C., Shen Y., Zhao J., Miao L. (2018). Evaluation of the in vitro Chemosensitivity and Correlation with Clinical Outcomes in Lung Cancer using the ATP-TCA. Anticancer. Agents Med. Chem..

[20] Karekla E., Liao W.J., Sharp B., Pugh J., Reid H., Quesne J.L., Moore D., Pritchard C., MacFarlane M., Pringle J.H. (2017). Ex Vivo Explant Cultures of Non-Small Cell Lung Carcinoma Enable Evaluation of Primary Tumor Responses to Anticancer Therapy. Cancer Res..

[21] Moon Y.W., Choi S.H., Kim Y.T., Sohn J.H., Chang J., Kim S.K., Park M.S., Chung K.Y., Lee H.J., Kim J.H. (2007). Adenosine triphosphate-based chemotherapy response assay (ATP-CRA)-guided platinum-based 2-drug chemotherapy for unresectable nonsmall-cell lung cancer. Cancer.

[22] Szulkin A., Otvös R., Hillerdal C.-O., Celep A., Yousef-Fadhel E., Skribek H., Hjerpe A., Székely L., Dobra K. (2014). Characterization and Drug Sensitivity Profiling of Primary Malignant Mesothelioma Cells from Pleural Effusions. BMC Cancer.

[23] Vinayanuwattikun C., Prakhongcheep O., Tungsukruthai S., Petsri K., Thirasastr P., Leelayuwatanakul N., Chanvorachote P. (2019). Feasibility Technique of Low-passage In Vitro Drug Sensitivity Testing of Malignant Pleural Effusion from Advanced-stage Non-small Cell Lung Cancer for Prediction of Clinical Outcome. Anticancer. Res..

[24] Inoue M., Maeda H., Takeuchi Y., Fukuhara K., Shintani Y., Funakoshi Y., Funaki S., Nojiri T., Kusu T., Kusumoto H. (2018). Collagen gel droplet-embedded culture drug sensitivity test for adjuvant chemotherapy after complete resection of non-small-cell lung cancer. Surg. Today.

[25] Shaw G.L., Gazdar A.F., Phelps R., Ilona Linnoila R., Ihde D.C., Johnson B.E., Oie H.K., Pass H.I., Steinberg S.M., Ghosh B.C. (1993). Individualized Chemotherapy for Patients with Non-Small Cell Lung Cancer by Prospective Identification of Neuroendocrine Markers and in Vitro Drug Sensitivity Testing. Cancer Res..

[26] Shaw G.L., Gazdar A.F., Phelps R., Steinberg S.M., Linnoila R.I., Johnson B.E., Oie H.K., Russell E.K., Ghosh B.C., Pass H.I. (1996). Correlation of in vitro drug sensitivity testing results with response to chemotherapy and survival: Comparison of non-small cell lung cancer and small cell lung cancer. J. Cell Biochem. Suppl..

[27] Shie M.Y., Fang H.Y., Kan K.W., Ho C.C., Tu C.Y., Lee P.C., Hsueh P.R., Chen C.H., Lee A.K., Tien N. (2023). Highly Mimetic Ex Vivo Lung-Cancer Spheroid-Based Physiological Model for Clinical Precision Therapeutics. Adv. Sci..

[28] Clohessy J.G., Pandolfi P.P. (2018). The Mouse Hospital and Its Integration in Ultra-Precision Approaches to Cancer Care. Front. Oncol..

[29] Hidalgo M., Amant F., Biankin A.V., Budinska E., Byrne A.T., Caldas C., Clarke R.B., de Jong S., Jonkers J., Maelandsmo G.M. (2014). Patient-derived xenograft models: An emerging platform for translational cancer research. Cancer Discov..

[30] Meijer T.G., Naipal K.A., Jager A., van Gent D.C. (2017). Ex vivo tumor culture systems for functional drug testing and therapy response prediction. Future Sci. OA.

[31] Kodack D.P., Farago A.F., Dastur A., Held M.A., Dardaei L., Friboulet L., von Flotow F., Damon L.J., Lee D., Parks M. (2017). Primary Patient-Derived Cancer Cells and Their Potential for Personalized Cancer Patient Care. Cell Rep..

[32] Papp E., Steib A., Abdelwahab E.M., Meggyes-Rapp J., Jakab L., Smuk G., Schlegl E., Moldvay J., Sarosi V., Pongracz J.E. (2020). Feasibility study of in vitro drug sensitivity assay of advanced non-small cell lung adenocarcinomas. BMJ Open Respir. Res..

[33] Kim S.Y., Lee J.Y., Kim D.H., Joo H., Yun M.R., Jung D., Yun J., Heo S.G., Ahn B., Park C.W. (2019). Patient-Derived Cells to Guide Targeted Therapy for Advanced Lung Adenocarcinoma. Sci. Rep..

[34] Roscilli G., De Vitis C., Ferrara F.F., Noto A., Cherubini E., Ricci A., Mariotta S., Giarnieri E., Giovagnoli M.R., Torrisi M.R. (2016). Human lung adenocarcinoma cell cultures derived from malignant pleural effusions as model system to predict patients chemosensitivity. J. Transl. Med..

[35] Higashiyama M., Oda K., Okami J., Maeda J., Kodama K., Imamura F., Minamikawa K., Takano T., Kobayashi H. (2010). Prediction of Chemotherapeutic Effect on Postoperative Recurrence by in Vitro Anticancer Drug Sensitivity Testing in Non-Small Cell Lung Cancer Patients. Lung Cancer.

[36] Kawamura M., Gika M., Abiko T., Inoue Y., Oyama T., Izumi Y., Kobayashi H., Kobayashi K. (2007). Clinical evaluation of chemosensitivity testing for patients with unresectable non-small cell lung cancer (NSCLC) using collagen gel droplet embedded culture drug sensitivity test (CD-DST). Cancer Chemother. Pharmacol..

[37] Higashiyama M., Kodama K., Yokouchi H., Takami K., Nakagawa H., Imamura F., Minamigawa K., Kobayashi H. (2001). Cisplatin-Based Chemotherapy for Postoperative Recurrence in Non-Small Cell Lung Cancer Patients: Relation of the in Vitro Chemosensitive Test to Clinical Response. Oncol. Rep..

[38] Wilbur D.W., Camacho E.S., Hilliard D.A., Dill P.L., Weisenthal L.M. (1992). Chemotherapy of Non-Small Cell Lung Carcinoma Guided by an in Vitro Drug Resistance Assay Measuring Total Tumour Cell Kill. Br. J. Cancer.

[39] Ajani J.A., Baker F.L., Spitzer G., Kelly A., Brock W., Tomasovic B., Singletary S.E., McMurtrey M., Plager C. (1987). Comparison between Clinical Response and in Vitro Drug Sensitivity of Primary Human Tumors in the Adhesive Tumor Cell Culture System. J. Clin. Oncol..

[40] Gilazieva Z., Ponomarev A., Rutland C., Rizvanov A., Solovyeva V. (2020). Promising Applications of Tumor Spheroids and Organoids for Personalized Medicine. Cancers.

[41] Rezk R., Marín-García R., Gad A.K.B. (2021). The Fibrillar Matrix: Novel Avenues for Breast Cancer Detection and Treatment. Engineering.

[42] Baker B.M., Chen C.S. (2012). Deconstructing the third dimension: How 3D culture microenvironments alter cellular cues. J. Cell Sci..

[43] Baker B.M., Trappmann B., Wang W.Y., Sakar M.S., Kim I.L., Shenoy V.B., Burdick J.A., Chen C.S. (2015). Cell-mediated fibre recruitment drives extracellular matrix mechanosensing in engineered fibrillar microenvironments. Nat. Mater..

[44] Nazemi M., Rainero E. (2020). Cross-Talk Between the Tumor Microenvironment, Extracellular Matrix, and Cell Metabolism in Cancer. Front. Oncol..

[45] ang M., McKay D., Pollard J.W., Lewis C.E. (2018). Diverse Functions of Macrophages in Different Tumor Microenvironments. Cancer Res..

[46] Sahai E., Astsaturov I., Cukierman E., DeNardo D.G., Egeblad M., Evans R.M., Fearon D., Greten F.R., Hingorani S.R., Hunter T. (2020). A framework for advancing our understanding of cancer-associated fibroblasts. Nat. Rev. Cancer.

[47] van Ooijen H., Verron Q., Zhang H., Sandoz P.A., Frisk T.W., Carannante V., Olofsson K., Wagner A.K., Sandström N., Önfelt B. (2025). A thermoplastic chip for 2D and 3D correlative assays combining screening and high-resolution imaging of immune cell responses. Cell Rep. Methods.

[48] Alsaed B., Smolander J., Laitinen H., Lin L., Bobik N., Lahtinen L., Räsänen M., Jansouz S., Peltonen K., Jokinen E. (2024). Ex Vivo Modeling of Precision Immuno-Oncology Responses in Lung Cancer. Sci. Adv..

